# The Calcium-Dependent Interaction of S100B with Its Protein Targets

**DOI:** 10.1155/2010/728052

**Published:** 2010-08-17

**Authors:** Danna B. Zimmer, David J. Weber

**Affiliations:** ^1^Department of Veterinary Pathobiology, College of Veterinary Medicine, Texas A & M University, MS 4467 College Station, TX 77843-4467, USA; ^2^Department of Biochemistry & Molecular Biology, University of Maryland School of Medicine, 108 N. Greene St. Baltimore, MD 21204, USA

## Abstract

S100B is a calcium signaling protein that is a member of the S100 protein family. An important feature of S100B and most other S100 proteins (S100s) is that they often bind Ca^2+^ ions relatively weakly in the absence of a protein target; upon binding their target proteins, Ca^2+^-binding then increases by as much as from 200- to 400-fold. This manuscript reviews the structural basis and physiological significance of increased Ca^2+^-binding affinity in the presence of protein targets. New information regarding redundancy among family members and the structural domains that mediate the interaction of S100B, and other S100s, with their targets is also presented. It is the diversity among individual S100s, the protein targets that they interact with, and the Ca^2+^ dependency of these protein-protein interactions that allow S100s to transduce changes in [Ca^2+^]_intracellular_ levels into spatially and temporally unique biological responses.

## 1. Introduction

**Figure 1:**
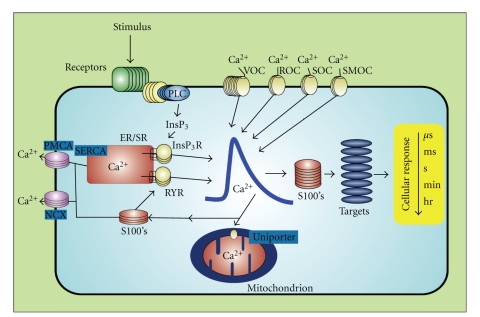
*S100s function as Ca^2+^-signaling proteins*. S100s bind and regulate protein targets as well as other Ca^2+^-signaling proteins in a Ca^2+^-dependent manner. S100s are distributed in a cell-specific manner to generate cell-type specific activities [1, 2, 10].

Ca^2+^ ions are important second messengers in all living cells [1]. Ca^2+^-binding proteins, including members of the calmodulin/troponin/S100 superfamily, maintain the integrity of the Ca^2+^ signal and transmit it in a temporally and spatially coordinated manner [2]. S100s were discovered in 1965 [3], and as with other EF-hand containing proteins, S100s also transduce changes in [Ca^2+^]_intracellular_ levels (i.e., [Ca^2+^]_i_) into cellular responses by binding Ca^2+^ (Table 1), changing conformation, and then interacting with and modulating the activity of other proteins (target proteins) (Figure 1). The amino acid homology between the current family members ranges from approximately 20% to 55% [4]. Because of the extensive amino acid homology between S100B and S100A1, the first two family members identified, early models predicted that individual members were functionally redundant and essentially interchangeable. As the number of family members discovered has increased and differences in their cellular/subcellular localization, physical properties, and target proteins have expanded, additional models have arisen with specific S100 family members having unique biological functions [5–12]. This multigenic family now contains up to twenty-one members (humans) whose phylogenetic distribution is restricted to higher chordates. Oligomerization properties, affinities for divalent metal ions (Ca^2+^, Zn^2+^, Cu^2+^), and posttranslational modifications also contribute to diversity among S100 family members [7, 12]. Furthermore, each cell/tissue expresses a unique subset of family members [11, 12]. Collectively, these findings support the view that S100s often confer cell type specificity to Ca^2+^ signal transduction pathways in cells and tissues [11–16].

The 3-dimensional structure of numerous S100 proteins has been solved by NMR and X-ray crystallography techniques in the apo-, Ca^2+^-bound, Zn^2+^-bound, drug-bound, and target protein bound states, and with the exception of calbindinD_9K_, S100s such as S100B are typically symmetric dimers with each subunit containing two EF-hand calcium-binding domains, although, some higher-order oligomeric states have also been detected and discussed [17–64]. The first EF-hand motif (EF1) in each subunit has fourteen rather than twelve residues and is termed the “pseudo-EF-hand” or “S100 EF-hand” [31, 65]. Another unique feature of the S100-hand (EF1) is that calcium-coordination is achieved via backbone carbonyl oxygen atoms rather than sidechain carboxylate oxygen atoms, and it typically binds Ca^2+^ rather weakly (K_D_> 0.5 mM). The second EF-hand (EF2) is termed the “typical EF-hand” since it has the same number of residues as most EF-hand Ca^2+^-binding proteins (i.e., twelve), and it exactly matches the consensus EF-hand in both sequence and three-dimensional structure [66]. As with the helix-loop-helix Ca^2+^ binding domains of other members of the EF-hand superfamily (i.e., calmodulin, troponin C, etc.), the second EF-hand of S100B coordinates Ca^2+^ via a backbone carbonyl oxygen at position 7, sidechain oxygen atoms from Asp/Glu at positions 1, 3, and 5, and via bidentate coordination from oxygen atoms from a Glu sidechain at position 12 [66]. Position 9 of the coordination scheme is occupied by a water molecule that is, in turn, hydrogen-bonded to a Glu sidechain oxygen atom from the pseudo-EF-hand. Another feature of S100B and several other S100s is that several of them bind Ca^2+^ ions relatively weakly, relative to [Ca^2+^]_i_, in the absence of a protein target (K_D_≥ 1 *μ*M). However, upon binding their full-length target protein, Ca^2+^-binding can then increase by as much as from 200- to 400-fold [60, 67]. This feature could allow for cells to contain high concentrations of some S100s, such as S100B (>1 *μ*M), without depleting [Ca^2+^]_i_ levels and “short-circuiting” Ca^2+^ oscillations necessary for signaling.

The large number of protein targets that S100B and other S100 family members bind and regulate provides yet another level of diversity/complexity to S100 signaling. Examination of the extensive lists of S100 target proteins provides important insights regarding S100 signaling [15, 68, 69]. First, S100s regulate a wide-range of cellular processes that includes energy metabolism, cytoskeleton organization, gene expression, and signal transduction pathways. Second, the list of *in vitro* target proteins for individual family members can be extensive, 23–25 different protein targets have been reported for S100B and S100A1 alone [5, 7, 13, 15, 68, 70]. Third, some target proteins interact with and have their activity modulated by multiple S100 family members suggesting functional redundancy among some family members. For example, both S100A1 and S100B interact with and activate aldolase A and aldolase C [71]. In contrast, S100A1 and S100B exhibit a Ca^2+^-dependent interaction with phosphoglucomtutase but this interaction results in differential effects on target protein activity: S100A1 stimulates phosphoglucomutase activity and S100B inhibits phosphoglucomutase activity [72]. Like phosphoglucomutase, both S100A1 and S100B bind glycogen phosphorylase a [73]. However, S100A1 inhibits and S100B has no effect on glycogen phosphorylase activity. Interestingly, S100A4, S100A1, and S100A6 interact with methionine aminopeptidase 2, but with different affinities [74]. The S100A6 (calcyclin) binding protein CACYBP also interacts with S100A1, S100A12, S100P, and S100B but not S100A4 [75]. And several S100 proteins (i.e., S100A1, S100A2, S100A4, S100A6, and S100B) bind to p53, p63, p73 [29, 76–80], and affect their biological functions [81–84]. Fifth, S100 activation of some target proteins is Ca^2+^-independent indicating that S100s sense changes in [Ca^2+^]_i_ levels and have important intracellular functions at both resting and stimulated [Ca^2+^]_i_ levels. Sixth, a subset of S100s have a conserved Zn^2+^-binding site that contributes to altered Ca^2+^-, target-, and in some cases, small molecule binding affinity *in vitro* [8, 20, 36, 40, 68, 85–99], which could potentially affect cellular functions. Seventh, the interaction of S100 family members with other family members as “target proteins” has been observed using yeast-two hybrid approaches [100–104]. Finally, S100 proteins may exert some of their effects via interaction with molecules other than proteins. For example, S100A8/S100A9 binds (poly) unsaturated fatty acids in a Ca^2+^-dependent manner [105, 106] and S100A1 binds IP_3_ (Baron, Coburn, and Zimmer, personal communication). New information regarding S100 family member specificity for individual target proteins and the structural motifs that mediate these interactions are detailed below. As discussed, the diversity among individual S100s, the protein targets that they interact with, and the Ca^2+^ dependency of these interactions are uniquely suited to conferring cell-type specificity to Ca^2+^-signaling pathways. 

### 1.1. The S100 Calcium-Switch

**Figure 2:**
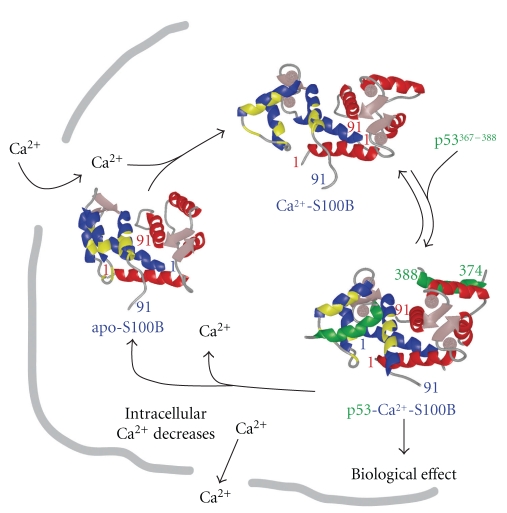
*The Ca^2+^-dependent S100-target protein interactions.* In red/blue are subunits of S100B (^dimer^K_D_< 500 pM [1, 17]) with regions shaded (yellow) for residues that bind targets such as p53^367-388^ (green), p53 (p53^321-346^, K_D_ = 24 ± 10 nM), or TRTK12 [10, 18].

**Figure 3:**
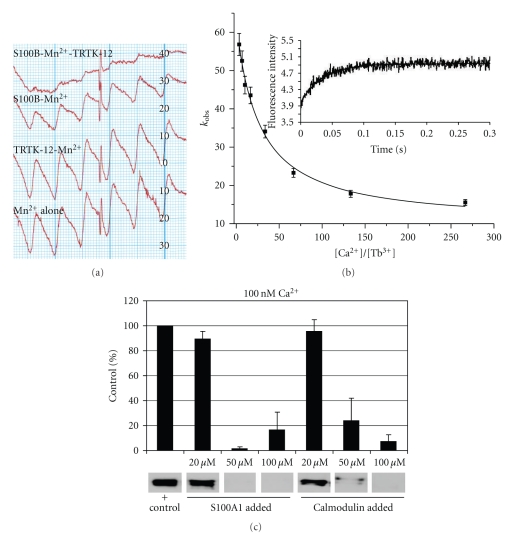
*Metal ion and target binding properties of S100 proteins.* (a) Binding studies with Mn^2+^ were completed since it is a good probe of the high affinity Ca^2+^ binding site on S100B (EF2) [113, 135]. Free Mn^2+^ was measured by electron paramagnetic resonance (EPR) in the absence and presence of S100B (+/− target peptide TRTK12) [34]. In all four traces, total [Mn^2+^] is identical (80 *μ*M) with the bottom trace (4th trace) showing the signal for total [Mn^2+^]. TRTK12 alone (1 mM) has no effect on the EPR signal (3rd trace), whereas, the addition of S100B (65 *μ*M) binds Mn^2+^ and reduces free [Mn^2+^] (2nd trace). The addition of the same amount of S100B (65 *μ*M) plus TRTK12 (1 mM, top trace) has the least free [Mn^2+^] and indicates that TRTK12 binding to S100B-Mn^2+^ enhances Mn^2+^ binding (compare traces 1 and 2). A similar effect was observed for SBi1 (unpublished) and for p53^367-388^ [109]. As for p53^367-388^ and SBi1, TRTK12 increased the affinity of S100B for Ca^2+^ in competition studies with Mn^2+^ and via stopped-flow kinetic measurements of ^Ca^k_off_ as monitored in competition with Tb^3+^. (b) Plot of the decrease in k_obs_ as a function of [Ca^2+^]/[Tb^3+^] as used to determine the off rate of Ca^2+^ from the 2nd EF-hand (EF2, ^Ca^k_off_). The k_obs_ values at each [Ca^2+^]/[Tb^3+^] ratio were calculated from kinetic traces of stopped-flow experiments where Tb^3+^ (syringe C) is mixed with S100B at varying Ca^2+^ concentrations (syringe A) and [Tb^3+^] signal is monitored as a function of time (*λ*_ex_ = 230 nm, *λ*_em_ = 545 nm). A ^Ca^k_off_ of 60 ± 8/sec was calculated from these experiments with S100B alone. When either TRTK12 or SBi1 is present, then the calculated ^Ca^k_off_ value for S100B is reduced to 5 ± 3/sec similar to that found for p53^367-388^ [109]. These studies demonstrated that TRTK12, p53^367-388^, or SBi1 increased the affinity of S100B for Ca^2+^ at least in part by decreasing ^Ca^k_off_. In (c), S100A1 was found to bind the full length ryanodine receptor (RyR) at 100 nM free calcium. Specifically, S100A1 competed full-length RyR1 away from agarose-linked CaM beads as judged by a decreased RyR1 band in an anti-RyR Western blot. Free CaM, a positive control, also competed the RyR away from CaM-linked beads [60, 67].

**Figure 4:**
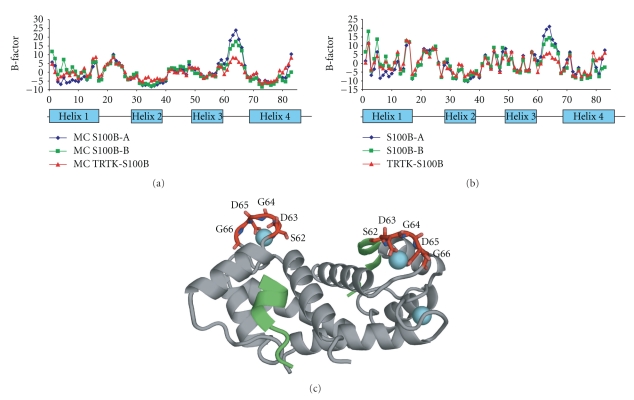
*B-factors for X-ray structures of TRTK12-Ca^2+^-S100B (2.0 Å) and Ca^2+^-S100B (1.5 Å) from the PI's laboratory. *(a) B-factors for backbone atoms for each subunit of Ca^2+^-S100B (blue, green) and for TRTK-Ca^2+^-S100B (red). (b) B-factors for sidechains with symbols as in (a). (c) Also shown is a ribbon diagram of the TRTK12-Ca^2+^-S100B structure with residues colored red in EF2 (residues 61–72), which display lower B-factors in the TRTK12-bound state (in panels (a) and (b)). These data are all published in Charpentier et al., 2010 [139].

Biophysical and structural biology techniques are used to study molecular determinants involved in Ca^2+^-dependent S100-target interactions. A comparison of apo- and Ca^2+^-bound S100B (B-Ca^2+^) indicates that the pseudo -EF-hand (EF1, ^Ca^K_D_> 350 *μ*M, Table 1) has minor structural changes when Ca^2+^ binds, whereas, the typical EF-hand (EF2, ^Ca^K_D_ = 56 ± 9 *μ*M, Table 1) has a large repositioning of several sidechain oxygen ligands during Ca^2+^ coordination (EF-2: D61, D63, D65, E67, D69, and E72) [23, 24, 26, 34, 78, 87, 107–109]. Much like S100A1, S100A4, and S100A5, Asp-61 and Asp-63, in positions 1 and 3 of the typical EF-hand, must rotate substantially to achieve a suitable Ca^2+^ binding orientation in S100B (Figures 2 and 4). That helix 4 is involved in the dimer interface explains why the entering helix 3 and not the exiting helix 4 moves significantly upon Ca^2+^ binding [23]. The conformational change, termed the “S100 Ca^2+^-switch”, is a feature unique to S100s and unlike other EF-hand proteins (i.e., calmodulin and troponin C) in which position 12 reorients the exiting helix upon Ca^2+^ binding [23, 26, 43, 44, 110, 111]. The conformational change of helix 3 involves breaking and forming hydrophobic contacts in several S100s with unique hydrophobic and/or hydrophilic residues becoming exposed for binding its protein target (Figures 2–4). However, S100A10 does not conform to other family members since it lacks a functional EF-hand Ca^2+^-binding domain, so its target protein interactions are all independent of Ca^2+^ [11, 112]. In several S100-target structures, much has been learned about specific target- and inhibitor-S100 interactions involving the “hinge (loop 2)”, the C-terminal loop, and the hydrophobic pocket involving helices 3/4 for several S100s (site 1), a second surface, nearby helix 4 and the Zn^2+^ site in S100B (i.e., site 2) was also discovered more recently in drug design studies aimed at inhibiting S100B [20, 21]. 

**Figure 5:**
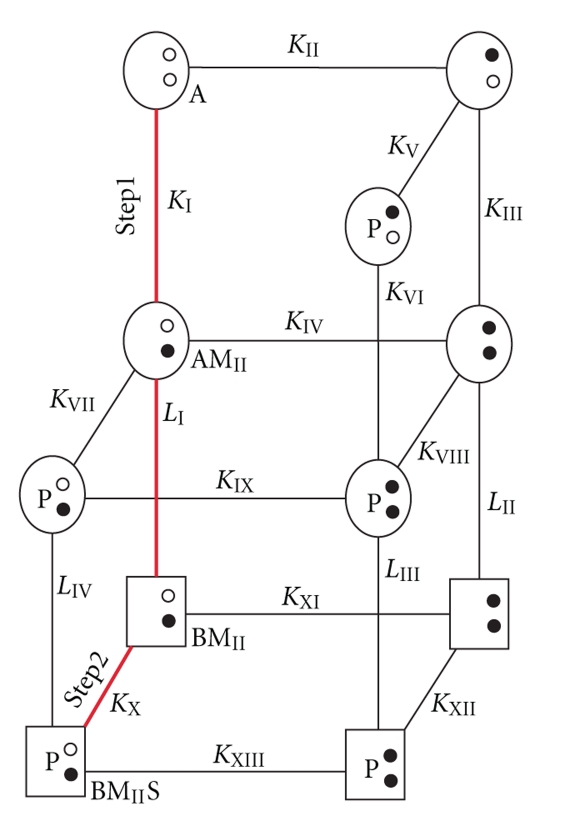
*Models for Ca^2+^-binding and then target-binding to an S100 protein.* (Top) A model for the Ca^2+^-dependent interaction of S100B with target proteins involves 13 equilibrium constants (K_I_ to K_XIII_), 11 states, and 4 conformational changes (L_I_–L_IV_) [109]. The most highly populated states and the predominant pathway are colored red; this is due to weak Ca^2+^ binding in the pseudo-EF-hand (site I), which greatly simplifies this model (see Scheme 1). Specifically, the binding of Ca^2+^ to the pseudo- and typical EF-hand in each S100B subunit is described by six states (A, AM_I_, AM_II_, AM_I_M_II_, BM_I_, and BM_II_), five equilibrium constants (K_I_ = [A][M]/[AM_I_], K_II_ = [A][M]/[AM_II_], K_III_ = [AM_I_][M]/[AM_I,II_], K_IV_ = [AM_II_][M]/[AM_I,II_], and K_XI_ = [BM_II_][M]/[BM_I,II_], two conformational changes (L_I_: AM_II_↔ BM_II_, L_II_: AM_I,II_↔ BM_I,II_) with corresponding rate constants, respectively, where A = S100B prior to the 90° reorientation of helix three of S100B, B = S100B after 90° reorientation of helix three, M_I_ = a Ca^2+^ ion bound to EF-hand I (pseudo-EF-hand), M_II_ = a Ca^2+^ ion bound to EF-hand II (typical EF-hand), M_I,II_ = Ca^2+^ ions bound to EF-hand I and EF-hand II. Upon the addition of p53 or another target (S), the model expands to 11 possible states, 13 dissociation constants, and four possible conformational changes. Whether additional equilibriums occur (K_XIV_, K_XV_, and K_XVI_) is considered in Scheme 1. (Bottom) In a second model (Scheme 1), state A is defined as the “closed” conformation observed in the apo-state (Figure 2), and state B is after a 90° reorientation of helix 3 termed the “open” conformation. In black, are states hypothesized to be populated. [A-M_II_]^‡^ and [B-M_II_]^‡^ represent short-lived intermediates, and L_1_ is the Ca^2+^-dependent conformational change involving helix 3 of S100B upon binding Ca^2+^ (Figure 2). Based on NMR relaxation rate data from the PI's lab [136], K_XIV_ highly favors state A. States are also considered via K_XV_ and K_XVI_ which result in B* states that represent an ensemble of dynamic structures, of which, only a subset fully coordinate Ca^2+^ as observed in X-ray structures [9]. It is hypothesized that K_XV_ favors the B*M_II_ state(s), whereas, K_XVI_ favors B-M_II_-S, explaining the apparent increase in Ca^2+^-binding affinity using equilibrium binding measurements that monitor free [metal ion] (Figure 3).

However, several important aspects pertaining to the Ca^2+^- and target-binding properties of S100s are not yet answered. With S100A5 and S100Z as exceptions (^Ca^K_D_~0.2 *μ*M, Table 1), dimeric S100s typically have a low affinity for binding Ca^2+^ *in vitro* (^Ca^K_D_≥ 1 *μ*M, EF2) [10, 124, 134](Table 1), leading some to classifying this family of proteins as “calcium-buffers” while others predicted that S100s only bound Ca^2+^ in locations where Ca^2+^ ion concentrations were relatively high (endoplasmic reticulum and the extracellular space). While low *in vitro* binding affinities were first thought to eliminate a role for S100 Ca^2+^-binding in the cytosol, where [Ca^2+^]_i_ typically oscillates between 0.1 and 1 *μ*M [1, 2], we now know that for many S100s, their Ca^2+^-binding affinity goes up significantly when its target protein is bound [34, 60, 109] (Figure 3). In fact, for S100A1 and the full-length ryanodine receptor (Figure 3(c)), the complex was detected at resting cytosolic [Ca^2+^]_i_ (i.e., at 100 nM) even though its Ca^2+^-binding affinity *in vitro* in the absence of target is ~300-fold higher (^Ca^K_D_ = 27 ± 2 *μ*M) [60] (Table 1). One explanation for the tightening Ca^2+^-binding affinity is that target binding induces a structural change to provide a more optimal Ca^2+^-coordination geometry. Such an explanation was ruled out for S100B when the X-ray structures of Ca^2+^-S100B (±TRTK12) and found that the Ca^2+^-coordination in both EF-hands (EF1, EF2) was indistinguishable despite the fact that ^Ca^k_off_ is ~20-fold slower when TRTK12 is bound [34]. Thus, the mechanism for “tightening” of Ca^2+^-binding with target bound cannot be explained by structural data alone. Interestingly, B-factors from X-ray structures (Figure 4) and ^15^N NMR relaxation rate studies of S100s have given us some indication that dynamic properties of S100 proteins are involved in the “tightening effect” (Figure 5). Thus, “induced-fit” versus “selected-fit” binding models for both the binding of Ca^2+^ and target to S100s have been considered. The underlying premise of the “selected-fit” model is that in the absence of target, the Ca^2+^-S100 complex observed in the X-ray structure is in equilibrium with a dynamic state(s) that has a lower Ca^2+^-binding affinity; when target protein binds to an Ca^2+^-S100 complex, the equilibrium shifts so “low affinity states” are eliminated when target binds to give less conformational exchange and an increase in measured Ca^2+^-binding (i.e., less free Ca^2+^, slower ^Ca^k_off_, Figure 3). Important for drug design, we found that S100B inhibitors can mimic target binding to also cause higher affinity Ca^2+^ binding (i.e., K_D_< 1 *μ*M). It is also now understood that weak Ca^2+^-binding for S100s in the *absence* of target may also turn out to be biologically relevant (Figure 1). Since most target-free S100s have low affinity for Ca^2+^, this allows numerous stable S100s to be at high concentrations in the cell (>1 *μ*M) without depleting [Ca^2+^]_i_ levels inside the cell and “short-circuiting” Ca^2+^ oscillations. Thus, several highly stable S100s can be “poised and ready” in any given cell for when their specific target(s) are expressed, as necessary for them to regulate numerous functions in mammalian cells (Figure 1) [109].

### 1.2. The S100 Model for Binding Calcium and Target

**Scheme 1:**
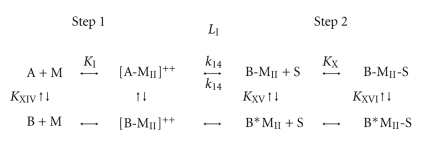


As a model system, Ca^2+^-dependent S100-target interactions are attractive, since S100s, in essence, have only one “functional” EF-hand (EF2, Figure 2) per subunit, which for S100B does not cooperate with the EF1 or EF1′/EF2′ of the other subunit in Ca^2+^-binding [10, 23, 31, 78, 87, 109]. This is very much unlike calmodulin (CaM) and troponin C (TnC), which have several functional and highly cooperative EF-hand Ca^2+^-binding domains [66]. Thus, a typical dimeric S100-target interaction is Ca^2+^-dependent and involves at least 11 possible states, 13 dissociation constants (K_1_–K_13_), four conformational changes (L_1_–L_4_), and the corresponding rate constants (k_1_–k_17_, k_−1_–k_−17_), per symmetric subunit [109] (Figure 5). As for Ca^2+^-binding, the conformational changes and the binding events for a single target are also symmetric and occur independently of those on the other subunit (i.e., without cooperativity), so only one S100 subunit needs to be considered in the thermodynamic scheme [10, 23, 31, 78, 87, 109]. At low [Ca^2+^] (Figure 5), the predominant kinetic pathway for target binding to S100s can be simplified to K_I_ (k_−1_/k_1_, step 1), L_1_ (k_−14_/k_14_), and K_X_ (k_−10_/k_10_, step 2) because an S100- or pseudo-EF-hand motif (EF1) binds Ca^2+^ about an order of magnitude more weakly than EF2 (K_I_ ≪ K_II_, i.e., K_II_~ 0.5 mM) [109]. However, in addition to a two-step K_I_ · L_1_ · K_X_ pathway for target binding, we will also consider three additional equilibriums resulting from conformational exchange prior to Ca^2+^ binding (K_XIV_), prior to target binding (K_XV_), as well as for the S100-target bound state (K_XIV_, Figure 5, Scheme 1). In step 1 (Scheme 1), structural data provides support for the “induced-fit” aspect of Ca^2+^ binding to S100B (i.e., A + Ca^2+^↔ [A − Ca^2+^]^‡^↔ B − Ca^2+^) since the apo-S100B (state A) has a patch of negatively charged residues comprising residues in the typical EF-hand (D65, E67, D69, and E72) sufficient for a fast bimolecular interaction with Ca^2+^ [23, 24]. Furthermore, no conformational exchange (R_ex_) has yet been observed in ^15^N-relaxation rate NMR studies of apo-S100B for any residues in helix 3 or in either of the Ca^2+^-binding loops that subsequently undergo structural transitions when Ca^2+^ is added [136]. However, this needs to be examined more rigorously for apo-S100B and for other apo-S100s using ZZ-exchange and relaxation dispersion NMR methods (off-resonance R_1*ρ*_, rcCPMG). Nonetheless, the lack of detectable R_ex_ in the apo-state currently provides an argument against the A ↔ B conversion (via K_XIV_) (Figure 5, Scheme 1, in green). In step 2, for target binding to an S100 (i.e., B −M_II_+ S ↔ B −M_II_− S, via K_X_), NMR data does exhibit R_ex_ for both Ca^2+^-S100B and Ca^2+^-S100A1 [59, 60, 77, 136, 137]; these preliminary data are consistent with a model in which target binding selects a competent conformation(s) from an ensemble of dynamic states (via K_XV_, i.e., “selected-fit” model [138]). The selected-fit hypothesis is also supported by the loss of exchange broadening upon binding of p53 and other targets to Ca^2+^-S100B [77, 113]. Further, the rate of Ca^2+^ dissociation (^Ca^k_off_) from EF2 decreases from 60/s to 7/s when p53 binds as measured by stopped-flow methods [109]; similar results were found when other targets, including an S100B inhibitor (SBi1) bound to Ca^2+^-S100B (Cannon and Weber, unpublished results). We now have X-ray crystal structures of S100B-Ca^2+^ and S100B-Ca^2+^-TRTK12 that show Ca^2+^ coordination is indistinguishable in the two complexes [34], whereas, elevated B-factors were observed for S100B-Ca^2+^ for residues in EF2 in the absence of bound target (Figure 4). One interpretation of the elevated B-factors is that there is conformational dynamics affecting EF2 in the absence of target, however, other explanations cannot be ruled out such as the possibility that lattice contacts are different in the two structures giving rise to the changes in B-factors [34]. Thus, it is important to further examine the dynamics of Ca^2+^-S100s, including EF2, directly by NMR to determine whether the decreased B-factors in EF2 (Figure 4) were due to a loss of conformational exchange as represented in Figure 5.

Additional evidence supporting Scheme 1 (Figure 5) is from stopped-flow experiments with S100-Ca^2+^ and S100B-Ca^2+^-target complexes [109]. Here, a fast kinetic-step at the earliest time points in stopped-flow traces has been observed (i.e., biphasic, Cannon and Weber, unpublished results), indicative of K_XV_. Also satisfying is that K_XV_ in Figure 5 provides a means for a single S100 protein to sample conformational space (i.e., at BM_II_), as may be necessary to bind more than one target protein, an observation made for several S100 proteins [10]. Structurally similar S100 proteins may also bind the same protein target (i.e., TRTK12), although k_off_ (i.e., k_−10_) in these cases usually varies due to specific differences in the binding site that give S100-target protein complexes unique conformations and hence varying “lifetimes” inside the cell (i.e., different ^Ca^k_off_ values). These issues regarding specificity are important and require further examination. It is also necessary to remember that one assumption in this model (Figure 5) is that binding of Ca^2+^ to EF1 is not significant. While this assumption is valid based on existing K_D_ values (and verified for S100B) [87]), weak Ca^2+^ binding to this EF-hand (EF1) in other S100s may slightly populate additional states, which together with cooperative binding effects observed in some cases [15] could complicate interpretations with this simple model (Figure 5).

### 1.3. S100 Family Members Exhibit Overlapping but Distinct Target Protein Binding Profiles

**Figure 6:**
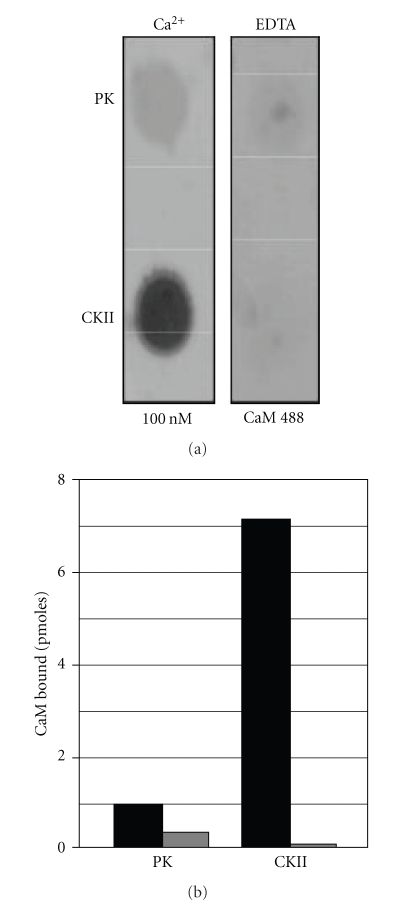
*Characterization of CaM-488 target protein binding*. Equimolar concentrations of CaM kinase II (CKII) phosphorylase kinase (PK) were immobilized on a PVDF membrane and incubated with 100 nM CaM-488 in the presence of Ca^2+^ or EDTA. Panel A contains a representative dot blot image. The histograms in Panel B represent the mean pmoles CaM-488 bound in the presence (black bars) and absence (gray bars) of Ca^2+^ assayed in triplicate in two independent experiments. Consistent with reported K_d_s and K_a_s, both targets exhibited Ca^2+^-dependent binding with the higher affinity target, CKII, binding more CaM-488 (7.0 pmoles) when compared to the lower affinity target PK (0.8 pmoles).

While structural, biochemical, and biophysical approaches yield important structural and mechanistic details, they cannot be used to screen simultaneously the interaction of multiple S100 family members with an extensive array of full-length target proteins. Therefore, we developed a quantitative assay that monitors the interaction of fluorophore labeled S100s with membrane-immobilized target proteins that could be used to efficiently identify/prioritize S100-target protein interactions and S100 domains for structural/mechanistic analyses. First, commercially available Alexa-Fluor 488-conjugated calmodulin (CaM-488) and two well-characterized calmodulin target proteins, calmodulin-dependent kinase II (CaM kinase II) and phosphorylase kinase, were used to determine if this methodology would yield data representative of published K_D_s and K_A_s (Figure 6). The interaction of CaM-488 with both target proteins was Ca^2+^ dependent. Immobilized CaM kinase II bound 5.4 pmoles CaM-488 in the presence of Ca^2+^ and 0.1 pmoles CaM-488 in the absence of Ca^2+^. Similarly, immobilized phosphorylase kinase bound 0.7 pmoles CaM-488 in the presence of Ca^2+^ and 0.3 pmoles CaM-488 in the absence of Ca^2+^. Furthermore, the 8-fold difference in bound CaM-488 (5.4 versus 0.7 pmoles) is in qualitative agreement with the higher affinity reported for CaM kinase II when compared to phosphorylase kinase [140, 141]. These data demonstrate the feasibility of using a membrane binding assay and Alexa-Fluor conjugated Ca^2+^-receptor proteins to monitor Ca^2+^ dependency of target protein interactions and to qualitatively compare the binding interaction.

**Figure 7:**
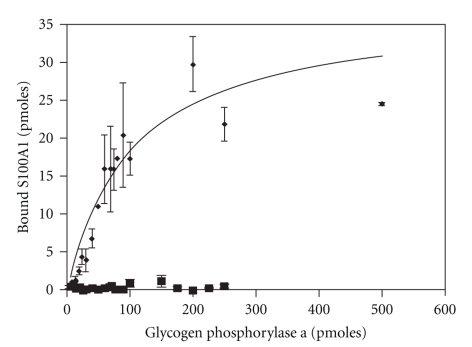
*S100A1-488 binding curves for glycogen phosphorylase a.* Membranes containing varying concentrations of glycogen phosphorylase were incubated in S100A1-488 in the presence (∙) or absence (■) of Ca^2+^. A standard curve of fluorescence intensity per mg of S100-488 was used to determine the experimental amount of labeled S100 per dot of target protein (*n * = 17).

**Figure 8:**
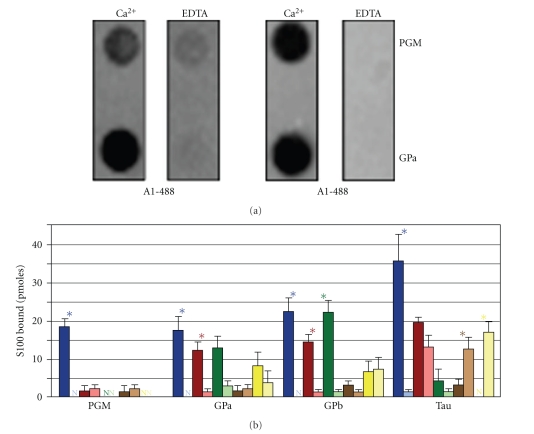
*Target protein binding profiles for S100 family members.* Membranes containing glycogen phosphorylase (a) (Gpa), glycogen phosphorylase (b) (Gpb), phosphoglucomutase (PGM), and tau (50 pmoles) were incubated in 100 nM Alexa Flour 488 labeled S100B (blue bars), S100A1 (red bars), S100P (green bars), S100A4 (brown bars), and S100A5 (yellow bars) in the presence (darker bars) or absence (lighter bars) of Ca^2+^. The histograms depict that the mean pmoles S100 bound ± the SEM and N's denote no detectable binding. Asterisks denote *P *≤  .05 between the ±Ca^2+^ conditions.

 To verify that this methodology could also be used to monitor S100-target protein interactions, the binding of S100A1-488 to a previously characterized target protein, glycogen phosphorylase a, was evaluated [73]. Consistent with results of previous gel overlay and affinity chromatography experiments, S100A1-488 exhibited Ca^2+^-dependent binding to immobilized glycogen phosphorylase a at all points (Figure 7). Furthermore, S100A1-488 binding was saturable with a B_max _ of 30.0 pmoles and EC_50_ for binding value of 37.5 pmoles. While this type of assay does not provide a K_D_, the B_max _ and EC_50_ can be used to compare S100-target protein interactions. Nonetheless, full-binding curves do not permit the simultaneous characterization of multiple S100s interacting with numerous target proteins. Therefore, two targets which exhibit differential interactions with S100A1 and S100B were used to determine if a single point assay like the one used in Figure 6 to characterize CaM target protein interactions would accurately reflect S100 target protein interactions. The quantity of target protein (50 pmoles) and probe concentration (100 nM S100A1-488 or S100B-488) were based on the EC_50_ (~40 pmoles) for glycogen phosphorylase a in 100 nM S100A1-488 (Figure 8). S100A1-488 and S100B-488 exhibited Ca^2+^-dependent binding to phosphoglucomutase as well as glycogen phosphorylase, and the range of binding was similar to that observed for CaM target proteins. Glycogen phosphorylase a and b bound similar amounts of S100A1-488 (12–15 pmoles) and S100B-488 (15–20 pmoles). In contrast, phosphoglucomutase bound 5-fold more S100B-488 (14 pmoles) than S100A1-488 (2 pmoles). This differential binding was undetectable in previous gel overlay and affinity chromatography experiments despite the differential effects of S100A1 and S100B on phospohglucomutase activity [142]. The insensitivity/inability of the overlay and affinity chromatography experiments to detect differential binding is most likely attributable to the high levels of receptor and/or ligand present. Altogether, these results demonstrate that a single point fluorometric assay can be used to quickly assess the relative affinity and Ca^2+^-dependency of S100-target protein interactions. 

Next, we compared the ability of different S100 family members (S100A1, S100A4, S100A5, and S100P) to interact with four previously reported S100B targets, glycogen phosphorylase a, glycogen phosphorylase b, tau, and phosphoglucomutase (Figure 8). As expected, all four target proteins bound S100B-488 (10–35 pmoles) in a Ca^2+^-dependent manner. While none of the other family members exhibited the same target protein binding profile as S100B, there did appear to be two distinct groups: one group (S100A1 and S100P) with profiles that were similar to and another group (S100A4 and S1005) that clearly distinct from S100B. Glycogen phosphorylase a and b bound similar levels of S100A1-488/S100P-488. However, phosphoglucomutase did not bind S100P-488 and 6-fold less S100A1-488 when compared to S100B-488. In addition, tau bound >10 fold less S100P-488 than S100B-488 and binding to S100A1-488 was Ca^2+^ independent. In the case of S100A4 and S1005, binding to all target proteins was below 10 pmoles. Finally, labeled S100A1, S100B, S100A4, and CaM (100 nM) did not bind to immobilized S100 family members (S100A1, S100B, S100A2, S100A4, S100A5, S100A11, and S100A13), calmodulin, or the negative control *α*-lactalbumin (75 pmoles) in the presence or absence of Ca^2+^ (data not shown). Collectively these data demonstrate that while there is extensive overlap among S100 family members with regard to the target proteins that they interact with, each S100 interacts with a unique compliment of target proteins. Nonetheless, there do appear to be family member-specific target proteins. For example, phospohglucomutase preferentially interacts with S100B. Furthermore, the relative affinity and Ca^2+^ dependency of the interaction can vary among family members. For example, tau is a target protein for multiple S100s including S100A1, S100A4, S100A5, and S100P, but only the interaction of tau with S100B is Ca^2+^-dependent.

### 1.4. The Linker Region Mediates Family Member-Specific Binding

The linker region, the amino acid sequence that links the two EF-hands, exhibits the greatest sequence diversity among family members and has been postulated to regulate family member-specific binding to target proteins such as phosphoglucomutase. Consistent with this hypothesis is the accessibility of this region to solvent in both the apo- and Ca^2+^-bound states of all family members for which 3D structures are available [10, 23, 57, 143]. To test this hypothesis, we checked the ability of a recombinant S100B-A1-B chimeric protein to bind to target proteins, in which the S100B linker region was replaced with comparable amino acid sequence from S100A1 (Figure 8). This chimeric protein was readily purified using similar procedures as used previously for S100B/S100A1, and the mutations to the hinge region did not exhibit any altered biochemical or biophysical properties tested. As anticipated, the interaction of the fluorophore-labeled chimeric protein with the S100A1/S100B targets glycogen phosphorylase a and b was indistinguishable from that of S100A1-488 and S100B-488, that is, Ca^2+^-dependent binding in the 10–25 pmole range. In contrast, phosphoglucomutase, a target protein that preferentially interacts with S100B, bound 2–4-fold less S100B-A1-B-488 when compared to S100B-488. Interestingly, the presence of the S100A1 linker region did not lower and/or reverse the Ca^2+^-dependent interaction with tau. Additional experiments will be needed to ascertain the linker region's contribution to other family-member specific target protein interactions as well as Ca^2+^-independent target protein binding. Nonetheless, this is the first demonstration that the linker region does confer family-specific binding for some Ca^2+^-dependent target proteins.

### 1.5. The Ca^2+^-Dependent Target Protein Binding Domain

**Figure 9:**
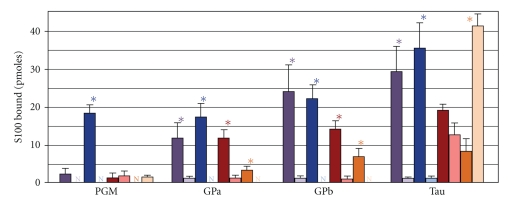
*Interaction of wild-type and mutant S100s with target proteins. *Membranes containing 50 pmoles glycogen phosphorylase a (Gpa), glycogen phosphorylase b (Gpb), phosphoglucomutase (PGM), and tau were incubated in 100 nM S100B-488 (blue bars), S100A1-488 (red bars), chimeric S100B-A1-B-488 (purple bars), or S100A1(F88/89A-W90A)-488 (orange bars) in the presence (darker bars) or absence (lighter bars) of Ca^2+^. The histograms depict that the mean pmoles S100 bound ± the SEM and N's denote no detectable binding. The asterisks denote *P *≤  .05, and the N's denote no detectable binding between the ±Ca^2+^ conditions.

Carboxyl terminal aromatic residues (Phe88, Phe89, Trp90) of S100A1 have been previously shown to regulate Ca^2+^-dependent interaction of S100A1 with the TRTK peptide, GFAP, and tubulin [72, 144, 145]. Analogous residues are found in several other members including S100B, S100A4, and S100A10 [78, 146–149]. To determine if these residues are obligatory for Ca^2+^-dependent target protein interactions, we examined the interaction of the S100A1 (F88/89A-W90A)-488 with four additional S100A1 target proteins (Figure 9). As anticipated, mutant S100A1 binding to all four target proteins was decreased by *∼*4-fold in the presence of Ca^2+^. Interestingly, there was a 4-fold increase in mutant S100A1 binding to tau in the absence of Ca^2+^. These results confirm that carboxy-terminal aromatic residues contribute to the Ca^2+^-dependent interaction of S100s with protein targets. However, it is not the only mechanism because not all family members that exhibit Ca^2+^-dependent target protein interactions have hydrophobic residues in their C-terminal extension [150]. 

## 2. Summary

In summary, S100 family members have a distinct role in intracellular Ca^2+^ signaling. The complement of S100s and S100 target proteins expressed in an individual cell allows that cell to transduce a universal change in [Ca^2+^]_i_ into a unique biological response. Furthermore, their unique metal binding properties and diverse lists of target proteins provide mechanisms for conferring Ca^2+^/metal sensitivity to cellular processes as well as integration and cross-talk among these processes. Thus, delineating S100-regulated processes in different cell types, ascertaining the relationship between intracellular and extracellular S100s, determining how S100-regulated processes are altered by Ca^2+^ dysregulation in disease states, and identifying that the molecular events involved are critical for understanding the function of this versatile protein family. The combination of structural, biochemical, molecular, cell biological, and *in vivo* techniques, which has been successful to date, will ultimately identify “inhibitors” of S100 function that can be used to normalize Ca^2+^ signaling in diseased cells.

## 3. Materials and Methods

### 3.1. Bacterial Expression Vectors for Wild-Type S100 Family Members

The pVex expression vector for human S100P was a generous gift of Dr. George Makhatadze [151]. Bacterial expression vectors for S100A1 and S100B have been previously described [152]. Plasmids encoding rat S100A4 (GenBank accession number AA997272) and S100A5 (GenBank accession number 1772854) were obtained through the IMAGE consortium library (Research Genetics, Huntsville, AL). The coding sequences were amplified using gene specific primers containing synthetic Nde I and Hind III sites at the 5′ and 3′ ends of the coding sequence, respectively. The nucleotide sequences for the primers were 5′-TTCCATATGGCGAGACCCTTGGAGGAG-3′ and 5′-CCCAAGCTTCACTTCTTCCGGGGCTCC-3′ (Lone Star Labs, Houston, TX) for S100A4, 5′-TTCCATATGGAGACTCCTCTTGAGAAG-3′ (Invitrogen, Carlsbad, CA) and 5′-CCCAAGCTTCACTTGTTGTCCTCTAAG-3′ (Lone Star Labs) for S100A5. Gene amplification was performed using an initial heat step (94°C, 5 minutes) followed by 30 cycles consisting of 1 minute at 94°C, 1 minute at 60°C, and 2 minutes at 68°C, and a final extension step of 7 minutes at 68°C. The resulting PCR products were subcloned into the TA cloning vector PCR2.1 (Invitrogen, Carlsbad, CA). The coding sequences were isolated from Nde I-Hind III digests of plasmid DNA and subcloned into pET21a^+^ (Novagen, San Diego, CA). The entire protein coding sequence was verified by DNA sequence analysis.

### 3.2. Bacterial Expression Vectors for Mutant S100 Proteins

The bacterial expression vector for the S100A1 triple point mutant (F88/89A-W90A) has been described previously [152]. A two-step PCR protocol followed by directional subcloning was used to generate the expression vector for the S100B-A1-B chimeric protein in which the amino acid sequence for the linker region in S100B (HFLEEIKEQ) was replaced with the S100A1 linker region (SFLDVQKDA). A sense oligonucleotide containing the amino terminal S100B sequence with an engineered Nde I restriction site (5′-CGCCATATGTCTGAACTCGAGAAAGCTG, Invitrogen) and a 3′ antisense oligonucleotide encoding the amino terminal half of the S100A1 linker region and an Xba I site (5′-CCTCTAGAAAGCTGCTAAGTTC, Invitrogen) were used to generate the 5′ half of the chimeric protein. The 3′ half of the chimeric protein was generated using the same template, a sense oligonucleotide encoding the carboxyl terminal half of the S100A1 linker region and an Xba I restriction enzyme site (5′-GCCATTTTCTAGACGTCCAGAAGGACGCGGAAGTTGTAGAC-3′, Integrated DNA Technologies, Coralville, IA) and an antisense oligonucleotide encoding the amino terminus of S100B with an engineered Hind III site (5′-CCCAAGCTTATTCATGTTCG, Intergrated DNA Technologies). The PCR program consisted of 30 cycles (1 minute denaturing at 94°C, 1 minute of annealing at 55°C, and 3 minutes of extension at 72°C). The resulting PCR products were ligated into the TA cloning vector PCR2.1. Purified insert DNA encoding the two halves of the chimeric protein was ligated together and subcloned into pET21a^+^ using the 5′ Nde I and 3′ Hind III restriction sites. Restriction enzyme digest and sequence analysis confirmed that the insert sequence encoded a protein with the appropriate amino acid sequence changes.

### 3.3. Expression/Purification of Recombinant S100 Proteins

Recombinant S100B, S100A1, and S100A1 triple point mutant were purified as previously described [152]. Minor modifications in this procedure were made to optimize purification for S100A4, S100A5, S100P, and the S100B-A1-B chimera. Unboiled bacterial lysates for S100A4, S100A5, and S100P were fractionated by ammonium sulfate precipitation. Prior to phenyl-Sepharose chromatography, solid ammonium sulfate was added up to 30% (w/v) to S100A4 [153] and up to 60% for S100P and S100A5. The unboiled S100B-A1-B lysate and the S100A4, S100A5, and S100P ammonium sulfate fractions were chromatographed on phenyl-Sepharose resin equilibrated with 50 mM Tris-Cl pH 7.4, 5 mM CaCl_2_, and 1 mM *β*-mercaptoethanol. The resin was washed with buffer containing high salt (50 mM Tris-Cl pH 7.4, 5 mM CaCl_2_, 1 mM *β*-mercaptoethanol, and 500 mM NaCl). Protein was eluted in 50 mM Tris-Cl, pH 7.4, 5 mM EDTA, and 1 mM DTT. Fractions containing S100 protein were identified by SDS-PAGE and pooled. The purity of the protein preparations was assessed by SDS-PAGE. Protein samples were considered to be >95% pure when only a single band of ~10,000 kDa was visible on the Coomassie-blue stained gel. 

### 3.4. Alexa Fluor Conjugation

All wild-type and mutant S100 proteins (5.0 mg/ml in PBS) were labeled with the photostable fluorophore, Alexa Fluor 488 (Molecular Probes, Eugene, OR), according to the manufacturer's recommendations. Prior to labeling, the pH was adjusted to ~8.3 with 1 M sodium bicarbonate and EDTA was added to a final concentration of 5.0 mM. Alexa Fluor 488 dye (1 mg) dissolved in DMSO was added to the protein sample. The reaction was incubated at room temperature for one hour in the dark with continuous stirring. Unconjugated dye was hydrolyzed by incubation of the solution overnight in the dark at 4°C. After dilution with 1-2 volumes of water, the pH of the solution was adjusted to 7.4 by the addition of 2 M Tris-Cl pH 7.4. Free dye was separated from protein conjugates by Ca^2+^-dependent phenyl-Sepharose chromatography as described for protein purification. Next, we determined if this methodology could be used to quantify the binding of S100 proteins to their various target proteins. Alexa Fluor 488 labeled S100A1, S100B, S100A4, S100A5, and S100P exhibited physical characteristics that were indistinguishable from unlabeled proteins including Ca^2+^-dependent binding to phenyl-Sepharose (data not shown).

### 3.5. S100-Target Protein Binding Assay

Glycogen phosphorylase a, glycogen phosphorylase b, phosphoglucomutase (PGM), and phosphorylase kinase were purchased from Sigma Chemical Company (St. Louis, MO). Bovine brain tau and calmodulin-dependent kinase II (CaM kinase II) were generous gifts of Gloria Lee (University of Iowa) and Tom Soderling (Vollum Institute, Portland, OR), respectively. Varying concentrations of the target proteins were immobilized on Immobilon-P PVDF membrane (Millipore) using a 96-well dot-blot apparatus (BioRad, Hercules, CA) per supplier's instructions. After a 30-minute incubation, wells were washed twice with three volumes of 20 mM Tris-Cl pH 7.4. The membrane was removed from the dot-blot apparatus, cut into strips, and incubated overnight at room temperature in 1 *μ*M Alexa Flour 488-labeled protein in 50 mM Tris-Cl, pH 7.4 with 200 mM NaCl (buffer A) containing either 5 mM EDTA or 1 mM CaCl_2_. The strips were rinsed three times in buffer A containing 5 mM EDTA or 1 mM CaCl_2_ and fluorescence quantified on a Fuji FLA-5000 Image Analysis System (Stamford, CT). A standard curve of fluorescence intensity versus pmoles of PVDF-immobilized S100-488 (or Cam-488) was used to determine the pmoles of labeled protein bound to that concentration of target protein (*n *≥ 2). Data points for each target protein concentration were averaged together to yield the mean pmoles of bound S100-488 ± SEM which was calculated for each target protein. The student's *t*-test was used to determine the statistical significance (*P *<  .05) of any measured differences between the means.

## Figures and Tables

**Table 1:** Dissociation of Ca^2+^ and Mn^2+^ from the EF-hand calcium-binding domains in wild-type and mutant S100 proteins.

S100 protein	EF1	EF2
*Ca^2+^ binding*		
S100B (wt)	>350 *μ*M^a,c^	56 ± 9 *μ*M^b,c^
S100B (E31A)	>500 *μ*M^a^	>500 *μ*M^a^
S100B (E72A)	480 ± 130 *μ*M^a^	>500 *μ*M^a^
S100B (E31A + E72A)	>2 mM^a^	>2 mM^a^
S100B (+p53)	—	20 ± 3 *μ*M^a^
S100B (E31A, +p53)	—	21 ± 7 *μ*M^a^
S100B (E72A, +p53)	—	18 ± 4 *μ*M^a^
S100B (E31A + E72A, +p53)	—	>300 *μ*M^a^
S100B (wt, +TRTK12)	—	12 ± 7 *μ*M^c^
S100A1 (wt)	—	27 ± 2 *μ*M^d^
S100A1 (wt, +TRTK12)	—	8 ± 3 *μ*M^e^
S100A2 (wt)	—	470 ± 50 *μ*M^f^
S100A3 (wt)	—	*∼*4 mM^g^
S100A4 (wt)	—	*∼*2.6 *μ*M^h^
S100A4 (wt, +p37)	—	*∼*0.2 *μ*M^h^
S100A5 (wt)	160 *μ*M^i^	*∼*0.2 *μ*M^i^
S100A6 (wt)	—	*∼*3.0 *μ*M^j^
S100A7 (wt)	—	*∼*1.0 *μ*M^k,l^
S100A11 (wt)	—	*∼*0.5 mM^m^
S100A12 (wt)	—	*∼*50 *μ*M^n^
S100A13 (wt)	*∼*400 *μ*M	*∼*8 *μ*M^o,p^
S100A16 (wt)	no binding	0.43 mM^q^
S100P (wt)	*∼*800 *μ*M	*∼*2.0 *μ*M^r^
S100Z (wt)	> 1 mM	*∼*0.2 *μ*M^s^

*Mn^2+^ binding*		
S100B (wt)	—	71 ± 12 *μ*M^a,c^
S100B (wt, +p53)	—	27 ± 4 *μ*M^a,c^
S100B (wt, +TRTK12)	—	6.0 ± 2.0 *μ*M^a,c^

^a^The value listed is from previously published papers [109, 113], so direct comparisons of binding constants using similar methods/conditions could be made (+/− target, Figure 3). Several others report binding constants using different methods and varying conditions for EF1 (200 *μ*M ≤K_D_≤ 500 *μ*M) and for EF2 (10 *μ*M ≤K_D_≤ 60 *μ*M) [58, 78, 86, 87, 114–120].

^b^The dissociation rate constant for wild-type S100B was determined via stopped-flow methods and is k_off_ = 60 ± 22 s^−1^. The off-rate together with the K_D_ enables the calculation of a macroscopic on-rate value of k_on_ = 1.1 × 10^6^ M^−1^ s^−1^ that includes calcium-association plus a large conformational change. The K_D_ value for the mutants was also determined using competition studies of Ca^2+^ with the respective Tb^3+^-bound S100B mutant in the absence and presence of p53 peptide. The dissociation constants together with the calcium off-rate values measured for the E31A and E72A mutants of 7.1 ± 3.7 s^−1^ and 6.8 ± 2.0 s^−1^, respectively, were sufficient to calculate on-rate values of 3.4 ± 2.0 × 10^6^ M^−1^ s^−1^ and 3.7 ± 1.3 × 10^6^ M^−1^ s^−1^ for the mutants [109, 113].

^c^From Charpentier et al. (2010) [34].

^d^From Wright et al. (2005) [61].

^e^From Wright et al. (2009) [59]. S100A1 has also been shown to bind the full-length ryanodine receptor at 100 nM free Ca^2+^ [60, 67].

^f^From Franz et al. (1998) [89].

^g^From Fritz et al. (1998). A tenfold weaker affinity was reported when purified under aerobic conditions [90, 121].

^h^From Dukhanina et al. (1998). A weaker affinity was reported under different conditions in Pedrocchi et al. (1994) when S100A4 was originally discovered [122, 123].

^i^From Schäfer et al. (2000). For a direct comparison of Ca^2+^ and Zn^2+^ binding to S100A5 to those of other S100 proteins (i.e., S100B, S100A2, S100A3, S100A4, S1006, and S10011), under identical conditions and Methods, also see Schäfer et al., (2000) [124].

^j^From Kuznicki and Filipek (1987) and Mani and Kay (1990). Kordowska et al. also measured Ca^2+^-binding for S100A6 under different conditions (^Ca^K_D_ *∼*18 *μ*M) and found that binding to the target caldesmon (CaD) increased the affinity of S100A6 for Ca^2+^ by approximately 6-fold [96, 125, 126]. Other measurements under higher salt and other varying conditions are also reported with weaker affinities for S100A6 [68, 124].

^k^From Schäfer et al. (2000) [124]. Weaker binding to Ca^2+^ has also been reported for this protein in other conditions [127].

^l^No data is available for S100A8/A9, and S100A10 does not bind Ca^2+^.

^m^From Allen et al. (1996) and Schäfer et al., (2000) [124, 128]. Note the affinity for Ca^2+^ increases by 10-fold upon the addition of a target molecule as found with other S100 proteins [128].

^n^From Dell'Angelica et al. (1994). Note that Zn^2+^-binding to S100A12 significantly increases Ca^2+^-binding affinity for this protein in the presence of Zn^2+^ (EF2: ^Ca^K_D_ = 40 nM, EF1: ^Ca^K_D_ = 15 *μ*M) [129].

^o^From Ridinger et al. (2000). This protein is unique among S100 family members in that it does not bind to the hydrophobic binding dye, TNS, upon the addition of Ca^2+^ [130].

^p^No data is yet available for S100A14, and there is no S10015 [131].

^q^From Sturchler et al. (2006). The value in the table is for human S100A16, mouse S100A16 bound one calcium too, only weaker (^Ca^K_D_ = 0.75 mM) [132].

^r^From Becker et al. (1992) and Gribenko et al., (1998) [92, 133]. In Gribenko et al., (1998), the effects of Mg^2+^ binding on Ca^2+^ affinity are also rigorously addressed.
